# Different Associated Factors of Subjective Cognitive Complaints in Patients With Early- and Late-Onset Parkinson's Disease

**DOI:** 10.3389/fneur.2021.749471

**Published:** 2021-11-23

**Authors:** Yi Xiao, Ruwei Ou, Tianmi Yang, Kuncheng Liu, Qianqian Wei, Yanbing Hou, Lingyu Zhang, Junyu Lin, Huifang Shang

**Affiliations:** Department of Neurology, Rare Disease Center, Laboratory of Neurodegenerative Disorders, National Clinical Research Center for Geriatric, West China Hospital, Sichuan University, Chengdu, China

**Keywords:** Parkinson's disease, subjective cognitive complaints, non-motor symptoms, early-onset Parkinson's disease (EOPD), mood disorder

## Abstract

**Background:** Subjective cognitive complaints (SCCs), which are associated with a higher risk of cognitive decline, are widespread in the patients with Parkinson's disease (PD). The previous studies have reported inconsistent factors related to SCCs in the patients with late-onset PD (LOPD), and there is limited information on SCCs in the patients with early-onset PD (EOPD).

**Objective:** We aimed to investigate the factors associated with SCCs in the drug-naïve patients with EOPD and LOPD without cognitive impairment.

**Methods:** This cross-sectional study included 332 drug-naïve patients with PD, among whom 134 were EOPD and 198 were LOPD. Motor and non-motor symptoms, such as global objective cognitive status, depression, anxiety, apathy, fatigue, sleep, rapid eye movement sleep behavior disorder, orthostatic hypotension, and excessive daytime sleepiness, were assessed.

**Results:** Twenty-five (18.66%) patients with EOPD and 49 (24.74%) patients with LOPD reported SCCs. A multivariate binary logistic regression analysis revealed that older age at onset [odds ratio (*OR*) = 1.24, *P* = 0.002], higher apathy score (*OR* = 1.13, *P* = 0.003), and lower scores in the visuospatial/executive abilities (*OR* = 0.25, *P* < 0.001) and memory (*OR* = 0.50, *P* = 0.024) domains of the Montreal Cognitive Assessment were associated with a higher risk of SCCs in the EOPD group. Higher apathy (*OR* = 1.06, *P* = 0.011) and anxiety (*OR* = 1.14, *P* < 0.001) scores were associated with SCCs in the LOPD group.

**Conclusion:** Subjective cognitive complaints are only associated with mood disorders in patients with LOPD. In addition, SCCs may reflect subthreshold cognitive impairment in the patients with EOPD.

## Introduction

The patients with Parkinson's disease (PD) experience many non-motor symptoms, such as cognitive impairment, mood disorders, and sleep disorders, which decrease the quality of life of patients (1). The cognitive status of patients with PD can be divided into three stages—normal cognition, PD with mild cognitive impairment (PD-MCI) (2), and PD with dementia (PDD) (3). Approximately 10–20% patients have PD-MCI at the time of PD diagnosis (4). Longitudinal studies have found that 13.8–27.6% patients with PD progress to PDD 5 years after diagnosis (5). Subjective cognitive complaints (SCCs) are defined as cognitive complaints reported by the patients and their caregivers (5). The prevalence of SCCs in patients with early PD without cognitive impairment has been reported to be 28.1–38.1% (6, 7). Moreover, several longitudinal studies have identified SCCs as a risk factor for PD-MCI and PDD (8–11). The risk of developing PD-MCI and PDD in the patients with SCCs and normal cognition has been shown to be 1.61 times higher than that in patients without SCCs during a 3–5-year period (8). Another study has reported a higher dementia conversion rate in the patients with SCCs and normal cognition than in those without SCCs (33 vs. 14.3%) during a 7.5-year follow-up (11). Since SCCs have great potential to predict cognitive impairment in PD, it is essential to explore factors associated with SCCs and manage them in the early stages of the disease.

The previous studies have revealed that SCCs are associated with mood disorders (6, 12, 13). For example, a study has shown that depression is associated with SCCs in the patients with an average disease duration of 6 years (12). In another study, anxiety was the only factor associated with SCCs (13). Further, a previous study found that depression, anxiety, and apathy were associated with SCCs (6). Recently, one study found that the patients with fatigue had a 4.97 times higher risk of SCCs than those without fatigue (14). Thus, limited information is available on the impact of non-motor symptoms of PD other than mood disorders and fatigue on SCCs.

In our previous study, we found that the patients with early-onset PD (EOPD) had a lower prevalence of issues in the context of perceptual problems/hallucinations, attention/memory, gastrointestinal, urinary, and sexual dysfunction, assessed by the Non-Motor Symptoms Scale, than those with late-onset PD (LOPD) (15). In another study, the patients with EOPD had a significantly slower rate of cognitive decline than those with LOPD (16). The previous studies have focused on SCCs in LOPD but not in EOPD. Only one study has reported that 5% patients with EOPD had SCCs (17).

Thus, in this study, we aimed to investigate the prevalence of SCCs among the drug-naïve patients with EOPD and LOPD without cognitive impairment and comprehensively analyze SCC-associated factors, such as motor and non-motor symptoms, such as fatigue, sleep, rapid eye movement sleep behavior disorder (RBD), excessive daytime sleepiness (EDS), orthostatic hypotension (OH), global objective cognitive status, depression, anxiety, and apathy.

## Method

### Patients

The patients with PD were consecutively recruited from the Department of Neurology, West China Hospital of Sichuan University between May 2018 and May 2021. All the patients with PD were diagnosed by movement disorder specialists using the 2015 version of the Movement Disorder Society Clinical Diagnostic Criteria for PD (18). In this study, only drug-naïve patients with PD were included to avoid the effect of dopaminergic drugs on patient cognition. EOPD was defined as disease onset before the age of 50 years (19). SCCs were assessed based on the response to Question one (“Over the past week have you had problems remembering things, following conversations, paying attention, thinking clearly, or finding your way around the house or in town?)” from the first section of the Movement Disorder Society Unified Parkinson's Disease Rating Scale (MDS-UPDRS); a score of ≥1 indicated SCCs (20). A higher score reflected more severe cognitive impairment in Question 1, with “No cognitive impairment,” “Slight impairment,” “Mild impairment,” “Moderate impairment,” and “Severe impairment” scored as 0, 1, 2, 3, and 4, respectively. The question was answered by the patients with PD. The exclusion criteria were diagnosis of objective cognitive impairment [PD-MCI level criteria (2) and diagnostic criteria for PDD (3)] and not completing the full assessments. Objective cognitive impairment was defined as a total Montreal Cognitive Assessment (MoCA) score < 26 (21). The local Ethics Committee of the West China Hospital of Sichuan University approved this study. A written informed consent was obtained from all the participants.

### Data Collection

Data on demographics and disease features, such as those on age, age at onset, sex, disease duration, years of education, body mass index (BMI), smoking history, and drinking history, were recorded. Global cognitive function was evaluated using the MoCA. The MoCA scores range from 0 to 30, with a higher score indicating better cognitive function (21). The severity of motor function was assessed using the MDS-UPDRS part III and Hoehn and Yahr (H&Y) stage. Non-motor symptoms were assessed using the specific scales. The Hamilton Depression Rating Scale (HAMD, 24 items, with scores ranging from 0 to 76) was used to assess depression, with a HAMD score > 20 indicating depression (22). The Hamilton Anxiety Rating Scale (HAMA, with scores ranging from 0 to 56) was used to measure anxiety, with a HAMA score > 14 indicating anxiety (23). The Fatigue Severity Scale (FSS, with scores ranging from 9 to 63) was used to evaluate fatigue, with a FSS score ≥36 indicated fatigue (24). The Lille Apathy Rating Scale (LARS, with scores ranging from −36 to 36) was used to measure apathy, with a LARS score ≥-16 indicating apathy (25). The sleep quality of patients with PD was assessed using the Parkinson's Disease Sleep Scale 2nd version (PDSS-2, with scores ranging from 0 to 60), and PD-related sleep dysfunction was defined as a PDSS-2 score ≥18 (26). EDS was assessed using the Epworth Sleepiness Scale (ESS, with scores ranging from 0 to 24), and EDS was defined as an ESS score ≥10 (27). RBD was assessed using the Rapid Eye Movement Sleep Behavior Disorder Screening Questionnaire and defined as a score of ≥5 (28). OH was defined as a drop in systolic blood pressure (BP) ≥20 mmHg and/or diastolic BP ≥10 mmHg (29). The patients were asked to lie down on the examination bed for 10 min before measuring the supine BP. Subsequently, the patients were asked to stand up and the BP was recorded at 1 and 3 min.

### Data Analysis

The demographics and clinical features are reported as frequencies (percent) and medians [interquartile range (IQR)] for the categorical and continuous variables, respectively. The Kolmogorov–Smirnov test was used to test the data for normality of distribution. Cohen's *d* was reported as effect size for continuous variables with 0.20 ≤ *d* < 0.50 indicating small effects, 0.50 ≤ *d* < 0.80 indicating moderate effects, and 0.80 < *d* indicating large effects. First, the univariate and multivariate logistic regression analyses were performed for the full sample. In univariate logistic regression, age; age at onset; sex; disease duration; education; MDS-UPDRS III scores; total score and each domain score of MoCA; scores of HAMD, HAMA, FSS, LARS, PDSS-2, and ESS; and presence or absence of RBD and OH were included as the independent variables. The presence or absence of SCCs was included as a dependent variable. Forward stepwise multivariate binary logistic regression was used to investigate the potential factors associated with SCCs. The factors with a *P*-value of ≤ 0.1 in the univariate analysis were included as independent variables, and presence or absence of SCCs was included as a dependent variable. Second, we divided the full sample into the EOPD and LOPD groups. The factors with a *P*-value of ≤ 0.1 in the univariate analysis of the EOPD group were included in logistic regression analysis for EOPD, and the same process of univariate and multivariate analysis was performed in LOPD. We used the Hosmer–Lemeshow test as statistical diagnostics to validate the regression model. All the analyses were performed using Statistical Package for the Social Sciences version 22.0. All statistical tests were two tailed, and statistical significance was set at *p* < 0.05.

## Results

### Baseline Demographic and Clinical Characteristics

A total of 134 and 198 drug-naïve patients with EOPD and LOPD, respectively, were recruited. Their demographic data and clinical features are listed in Table 1. A total of 25 (18.66%) patients with EOPD and 49 (24.74%) patients with LOPD reported SCCs. In the EOPD group, the patients with SCCs were older (*P* = 0.007, Cohen's *d* = 0.64), had older age at onset (*P* = 0.004, Cohen's *d* = 0.70), and had a lower education level (*P* = 0.027, Cohen's *d* = 0.51) than those without SCCs. In addition, the patients with EOPD with SCCs had a significantly lower score in the MoCA domains of visuospatial/executive abilities (*P* < 0.001, Cohen's *d* = 1.07) and memory (*P* = 0.043, Cohen's *d* = 0.46) and lower total MoCA score (*P* = 0.001, Cohen's *d* = 0.84) than those without SCCs. Furthermore, the patients with EOPD with SCCs had significantly higher FSS (*P* = 0.012, Cohen's *d* = 0.59), PDSS-2 (*P* = 0.007, Cohen's *d* = 0.66), LARS (*P* = 0.005, Cohen's *d* = 0.70), HAMD (*P* = 0.003, Cohen's *d* = 0.72), and HAMA (*P* = 0.018, Cohen's *d* = 0.56) scores than those without SCCs (Table 2).

**Table 1:** Demographic and clinical features of the patients with EOPD and LOPD.

	**All (*n* = 332)**	**EOPD (*n* = 134)**	**EOPD with SCC (*n* = 25, 18.66%)**	**EOPD without SCC (*n* = 109, 81.34%)**	**LOPD (*n* = 198)**	**LOPD with SCC (*n* = 49, 24.74%)**	**LOPD without SCC (*n* = 149, 75.25%)**
Age (years)	56.30 (46.70–65.50)	44.72 (39.14–48.15)	48.24 (44.64–49.14)	43.68 (38.45–47.41)	64.19 (57.43–70.91)	63.93 (57.92–71.82)	64.39 (57.30–70.36)
Male	178 (53.61%)	73 (54.48%)	13 (52.00%)	60 (55.05%)	105 (53.03%)	26 (53.06%)	79 (53.02%)
Age at onset (years)	54.39 (45.01–64.08)	43.06 (37.15–46.75)	47.14 (43.82–47.75)	41.91 (35.98–45.94)	61.93 (55.96–69.11)	61.19 (57.09–70.11)	62.31 (55.88–68.79)
Disease duration (years)	1.45 (0.75–2.40)	1.51 (0.91–2.76)	1.33 (0.82–1.93)	1.74 (0.91–3.02)	1.37 (0.69–2.24)	1.74 (0.75–3.01)	1.27 (0.67–2.07)
Education (years)	12 (9–16)	12 (9–16)	9 (8.5–13.5)	12 (9–16)	12 (9–15)	12 (9–15)	12 (9–15)
MDS-UPDRS III	21 (14–29)	19 (11–28)	20 (14.5–29)	19 (11–28)	22 (16–29)	28 (16–37)	22 (15–27)
H&Y stage	2 (1.5–2)	2 (1.5–2)	2 (1.5–2)	2 (1.5–2)	2 (2–2)	2 (2–2.5)	2 (2–2)
BMI	23.08 (20.98–25.20)	22.59 (20.76–24.76)	22.89 (21.07–24.73)	22.58 (20.70–24.87)	23.65 (21.33–25.39)	24.28 (21.44–26.18)	23.45 (21.30–25.19)
Smoking	87 (26.20%)	40 (29.85%)	8 (32.00%)	32 (29.36%)	47 (23.73%)	9 (18.37%)	38 (25.50%)
Drinking	98 (29.52%)	34 (25.37%)	3 (12.00%)	31 (28.44%)	64 (32.32%)	9 (18.37%)	55 (36.91%)
MoCA	27 (26–28)	27 (26–29)	26 (26–27)	28 (27–29)	27 (26–28)	26 (26–27)	27 (26–28)
Visuospatial/executive abilities	5 (4–5)	5 (4–5)	4 (3–4)	5 (4–5)	5 (4–5)	5 (4–5)	4 (4–5)
Naming	3 (3–3)	3 (3–3)	3 (3–3)	3 (3–3)	3 (3–3)	3 (3–3)	3 (3–3)
Attention	6 (6–6)	6 (6–6)	6 (6–6)	6 (6–6)	6 (6–6)	6 (6–6)	6 (6–6)
Language	3 (2–3)	3 (3–3)	3 (2.5–3)	3 (3–3)	3 (2–3)	3 (2–3)	3 (2–3)
Abstraction	1 (1–2)	1 (1–2)	1 (1–2)	1 (1–2)	1 (1–2)	1 (1–2)	1 (1–2)
Memory	4 (3–5)	4 (3–5)	3 (3–4)	4 (3–5)	4 (3–4)	3 (3–4)	4 (3–5)
Orientaion	6 (6–6)	6 (6–6)	6 (6–6)	6 (6–6)	6 (6–6)	6 (6–6)	6 (6–6)
FSS	23 (9–43)	29.5 (9–43)	42 (24.5–48.5)	24 (9–41)	19.5 (9–43)	35 (15–48)	17 (9–40)
PDSS−2	5 (3–10)	5 (2–9)	8 (3.5–13.5)	4 (2–7)	6 (3–10.25)	9 (5–16)	5 (3–10)
RBD	46 (13.85%)	10 (7.46%)	4 (16.00%)	6 (5.50%)	36 (18.18%)	10 (20.41%)	26 (17.45%)
LARS	−29 (−33 to −24)	−28 (−33 to −24)	−26 (−28.5 to −19.5)	−30 (−33 to −24)	−30 (−33 to −24)	−26 (−31.5 to −18)	−30 (−34 to −25)
HAMD	5 (1.25–9)	5 (2–10)	4.5 (11–15)	5 (2–9)	4 (1–8)	7 (4–15.5)	3 (1–7)
HAMA	5 (2–8)	5 (2–9)	8 (3.5–13)	4 (2–7)	4 (1–8)	7 (5–13.5)	3 (0–7)
ESS	3 (1–7)	4 (2–6)	4 (3–8.5)	4 (1–6)	3 (1–8)	3 (1–8)	3 (2–8)
Orthostatic hypotension	19 (5.72%)	7 (5.22%)	2 (8.00%)	5 (4.59%)	12 (6.06%)	4 (8.16%)	8 (5.37%)

*EOPD, early-onset Parkinson's disease; LOPD, late-onset Parkinson's disease; SCC, subjective cognitive complaints; H&Y stage, Hoehn and Yahr stage; MDS-UPDRS-III, Movement Disorder Society Unified Parkinson's Disease Rating Scale part III; BMI, body mass index; MoCA, Montreal Cognitive Assessment; HAMD, Hamilton Depression Rating Scale; HAMA, Hamilton Anxiety Rating Scale; FSS, Fatigue Severity Scale. LARS: Lille Apathy Rating Scale. PDSS-2: Parkinson's Disease Sleep Scale 2nd version. EDS: Excessive daytime sleepiness; RBD, rapid eye movement sleep behavior disorder; BMI, body mass index*.

**Table 2:** Factors associated with SCCs in the patients with EOPD and LOPD in univariate logistic regressions.

	**EOPD**			**LOPD**		
	**OR**	**95%CI**	***p*-value**	**OR**	**95%CI**	***p*-value**
**Demographic features**						
Age (years)	1.13	1.04–1.24	0.007	1.01	0.97–1.05	0.582
Male	1.13	0.47–2.70	0.783	1.00	0.52–1.91	0.996
Age at onset (years)	1.17	1.05–1.29	0.004^*^	1.01	0.96–1.05	0.824
Disease duration (years)	0.74	0.51–1.09	0.128	1.32	1.02–1.71	0.033^*^
Education (years)	0.87	0.76–0.98	0.027^*^	1.03	0.94–1.12	0.574
MDS-UPDRS III	1.01	0.98–1.05	0.474	1.05	1.02–1.08	0.003^*^
H&Y stage	1.74	0.74–4.06	0.204	2.59	1.22–5.53	0.014^*^
BMI	1.04	0.90–1.21	0.599	1.07	0.97–1.19	0.161
Smoking	1.13	0.44–2.89	0.795	0.66	0.29–1.48	0.311
Drinking	0.34	0.10–1.22	0.100	0.39	0.17–0.85	0.019^*^
**Non-motor symtoms**						
MoCA	0.46	0.29–0.73	0.001^*^	0.81	0.62–1.06	0.131
Visuospatial/executive abilities	0.32	0.18–0.56	< 0.001^*^	1.39	0.88–2.17	0.156
Naming	0.64	0.21–1.96	0.433	0.318	0.69–3.17	0.318
Attention	0.92	0.32–2.68	0.880	0.71	0.36–1.42	0.337
Language	1.08	0.43–2.69	0.875	0.92	0.54–1.55	0.747
Abstraction	0.66	0.30–1.44	0.292	0.80	0.47–1.36	0.408
Memory	0.62	0.39–0.99	0.043^*^	0.73	0.53–1.00	0.049^*^
Orientaion	0.43	0.10–1.84	0.254	0.84	0.34–2.06	0.700
FSS	1.04	1.01–1.06	0.012^*^	1.03	1.01–1.05	0.001^*^
PDSS-2	1.09	1.02–1.15	0.007^*^	1.06	1.01–1.11	0.011^*^
RBD	3.27	0.85–12.61	0.085	1.21	0.54–2.74	0.642
LARS	1.09	1.03–1.15	0.005^*^	1.09	1.04–1.13	< 0.001^*^
HAMD	1.11	1.04–1.19	0.003^*^	1.14	1.08–1.21	< 0.001^*^
HAMA	1.08	1.01–1.15	0.018^*^	1.17	1.10–1.25	< 0.001^*^
ESS	1.08	0.98–1.18	0.113	1.00	0.93–1.08	0.937
Orthostatic hypotension	1.809	0.33–9.91	0.495	1.57	0.45–5.45	0.480

*Results of univariate logistic regressions*.

* *p < 0.05. EOPD, early-onset Parkinson's disease; LOPD, late-onset Parkinson's disease; SCCs, subjective cognitive complaints; H&Y stage, Hoehn and Yahr stage; MDS-UPDRS-III, Movement Disorder Society Unified Parkinson's Disease Rating Scale part III; BMI, body mass index; MoCA, Montreal Cognitive Assessment; HAMD, Hamilton Depression Rating Scale; HAMA, Hamilton Anxiety Rating Scale; FSS, Fatigue Severity Scale; LARS, Lille Apathy Rating Scale; PDSS-2, Parkinson's Disease Sleep Scale 2nd version; EDS, Excessive daytime sleepiness; RBD, rapid eye movement sleep behavior disorder; BMI, body mass index*.

Meanwhile, the patients with LOPD with SCCs had significantly longer disease duration (*P* = 0.033, Cohen's *d* = 0.36), higher MDS-UPDRS III scores (*P* = 0.003, Cohen's *d* = 0.52), higher H&Y stage (*P* = 0.014), and lower rate of drinking (*P* = 0.019) than those without SCCs. Further, the patients with LOPD with SCCs had a lower score in the MoCA domain of memory (*P* = 0.049, Cohen's *d* = 0.33) than those without SCCs. Moreover, the patients with LOPD with SCCs had higher FSS (*P* = 0.001, Cohen's *d* = 0.55), PDSS-2 (*P* = 0.011, Cohen's *d* = 0.46), LARS (*P* < 0.001, Cohen's *d* = 0.81), HAMD (*P* < 0.001, Cohen's *d* = 0.90), and HAMA (*P* < 0.001, Cohen's *d* = 0.95) scores than those without SCCs (Table 2).

### Relationship Between Non-motor Symptoms and SCCs in the Patients With EOPD and LOPD

We first conducted a multivariate logistic analysis of the whole sample. The factors with a *P*-value of ≤ 0.1, such as age at onset; drinking; MoCA memory score; scores of LARS, HAMD, FSS, PDSS-2, HAMA, and MDS-UPDRS-III; age; and sex, were included in the multivariate logistic analysis of all the included patients with PD. SCCs were associated with older age at onset [odds ratio (*OR*) = 1.03, 95% *CI* = 1.00–1.05, *P* = 0.029], non-drinking history (*OR* = 0.37, 95% *CI* = 0.18–0.78, *P* = 0.009), lower memory scores (*OR* = 0.66, 95% *CI* = 0.49–0.88, *P* = 0.005), higher LARS score (*OR* = 1.05, 95% *CI*, 1.01–1.09, *P* = 0.013), and higher HAMD score (*OR* = 1.09, 95% *CI* = 1.04–1.15, *P* = 0.001) (Supplementary Material). No interaction effects were found between the disease subtypes and other independent variables in the additional logistic regression model.

We then analyzed the EOPD and LOPD subgroups separately. The factors with a *P*-value of ≤ 0.1, such as age at onset; MoCA score of visuospatial/executive abilities; MoCA score of memory; scores of LARS, FSS, PDSS-2, HAMD, and HAMA; education; RBD; drinking; age; and sex, were included in the multivariate binary logistic regression analysis of the patients with EOPD. The multivariate binary logistic regression analysis showed that an older age at onset (*OR* = 1.24, 95% *CI* = 1.08–1.43, *P* = 0.002) and a higher LARS score (*OR* = 1.13, 95% *CI* = 1.04–1.22, *P* = 0.003) were related to SCCs in the patients with EOPD. Moreover, lower scores in the MoCA domains of visuospatial/executive abilities (*OR* = 0.25, 95% *CI* = 0.13–0.49, *P* < 0.001) and memory (*OR* = 0.50, 95% *CI* = 0.27–0.91, *P* = 0.024) were associated with SCCs in the patients with EOPD (Table 3). The factors with a *P*-value of ≤ 0.1, such as MoCA memory score; scores of LARS, HAMA, FSS, PDSS-2, HAMD, and MDS-UPDRS-III; disease duration; drinking; and sex, were included in the multivariate binary logistic regression analysis of patients with LOPD. Finally, higher scores of LARS (*OR* = 1.06, 95% *CI* = 1.01–1.10, *P* = 0.011) and HAMA (*OR* = 1.14, 95% *CI* = 1.06–1.22, *P* < 0.001) were associated with SCCs in the patients with LOPD (Table 4).

**Table 3:** Correlative clinical factors of SCCs in the patients with EOPD.

	**OR**	**95%CI**	***p*-value**
Age at onset	1.24	1.08–1.43	0.002
**MoCA**			
Visuospatial/executive abilities	0.25	0.13–0.49	< 0.001
Memory	0.50	0.27–0.91	0.024
LARS	1.13	1.04–1.22	0.003

*EOPD, early-onset Parkinson's disease; SCCs, subjective cognitive complaints; MoCA, Montreal Cognitive Assessment; LARS, Lille Apathy Rating Scale*.

**Table 4:** Correlative clinical factors of SCCs in the patients with LOPD.

	**OR**	**95%CI**	***p*-value**
LARS	1.06	1.01–1.10	0.011
HAMA	1.14	1.06–1.22	< 0.001

*LOPD, late-onset Parkinson's disease; SCCs, subjective cognitive complaints; LARS, Lille Apathy Rating Scale; HAMA, Hamilton Anxiety Rating Scale*.

## Discussion

This large sample size study included the patients with EOPD and LOPD without cognitive impairment and adjusted for the most comprehensive independent factors of SCCs. To our knowledge, this is the first study to focus on the factors related to SCCs in EOPD. We found that SCCs are common in the patients with EOPD (18.66%), but less frequent than in patients with LOPD (24.74%). The EOPD and LOPD groups had different SCC-related factors. Older age at onset, higher apathy scores, and lower scores in the MoCA domains of visuospatial/executive abilities scores and memory were associated with SCCs in the patients with EOPD. In contrast, higher apathy scores and higher anxiety scores were associated with SCCs in the patients with LOPD. SCCs are associated with an increased risk of PD-MCI and PDD (8–11). Differences in the factors associated with SCCs between the patients with EOPD and LOPD identified in our study will enhance our understanding of SCCs in PD and, thus, help in clinical practice.

In our study, the rate of SCCs in the EOPD group (18.66%) was higher than that previously reported (5%) (17). In a previous study, SCCs were defined as reporting “moderate memory loss with disorientation and moderate difficulty handling complex problems” or worse cognitive problems. However, in this study, SCCs were defined as “impairments appreciated by the patient or caregiver with no concrete interference with the patient's ability to carry out normal activities and social interactions” or worse cognitive dysfunction. The definition of SCCs in our study and that in the previous studies was different. Thus, the low incidence of SCCs in the previous study might be because the patients reporting slight intellectual impairment were excluded (17). In addition, in the previous study, the patients with a Mini-Mental State Examination score of ≤ 23 were excluded, while in our study, the patients with a MoCA score of < 26 were excluded (17). The prevalence of SCCs in LOPD in our study (24.74%) was similar to that reported previously (28.1%) (6). In a large study of adults aged ≥45 years, the prevalence of SCCs increased with age (30). In a previous study, older age at onset contributed to cognitive decline in the patients with LOPD (31). With little research focusing on SCCs in middle-aged adults, more attention should be paid to this patient group in the future.

The patients with EOPD and LOPD had different patterns of alterations in emotion and cognition-related circuits compared with their age-matched healthy control in the previous studies (32, 33). In EOPD, decreased cerebral regional homogeneity (ReHo) values were found in parts of the frontal and left temporal regions (32). In LOPD, increased ReHo values were found in the left angular gyrus (32). Another study found increased ReHo in the left inferior temporal gyrus in the patients with EOPD and decreased ReHo values in the left insula in patients with LOPD (33). In our previous study, the patients with EOPD and LOPD showed different patterns of alteration in functional connectivity in the corticostriatal and cerebellostriatal loops (34). These different brain network alterations between EOPD and LOPD may contribute to the differences in SCC-related factors in the patients with EOPD and LOPD.

In patients with EOPD, lower scores in the MoCA domains of visuospatial/executive abilities and memory were related to SCCs. Visuospatial and executive abilities and memory were found to be the most commonly affected cognition domains in EOPD (35). Up to 72 and 65% of patients with EOPD had impairments in visuospatial and executive function and memory, respectively (scores < 1 SD or ≤ 20th percentile below the normal) (35). This indicates that SCCs may reflect subthreshold impairments in visuospatial and executive function and memory in the patients with EOPD, but the impairments did not meet the MCI criteria and impact the daily life of patients. However, further prospective longitudinal studies are needed to confirm this finding. Subjective memory complaints (SMCs) predicted PDD in the patients with PD-MCI (36). Therefore, memory-related SCCs in patients with PD should be brought to the forefront.

In this study, we found that anxiety was associated with a higher risk of SCCs in the patients with LOPD, which is consistent with the findings of some previous studies (6, 13). In the MCI group, those who reported SMC had increased odds of experiencing anxiety compared with those who did not report SMC (37). Among the patients with PD without dementia, those with anxiety performed worse in the executive function test than those without anxiety (38), and those with anxiety had a three times higher risk of MCI than those without anxiety (39). These studies revealed a close association between anxiety, SCCs, and cognitive decline.

We also found that apathy was a factor related to SCCs in the LOPD and EOPD groups. There is a lack of studies on apathy in EOPD, but several studies have been conducted in the patients with LOPD. A longitudinal study showed in the patients with LOPD, those with apathy at baseline had significantly lower global cognitive and executive function scores comparing with those without apathy. After a median follow-up of 18 months, the patients with LOPD with apathy had a significantly higher rate of conversion to dementia than those without apathy (40). Decreased gray matter (GM) density of the anterior cingulate cortex (ACC) was found in the patients with LOPD with SCCs compared with that in those without SCCs (41). Decreased GM density in the right ACC was also found in the patients with LOPD with apathy compared with that in those without apathy (42). It seems that damage to the ACC may also be a common mechanism for apathy and SCCs in the patients with EOPD and LOPD. However, functional MRI studies are required to confirm this hypothesis.

This study has several limitations. First, a single question evaluating SCCs was less comprehensive than a screening scale. However, SCCs screening through a single question has been confirmed as a risk factor for PD-MCI (8) which supports the clinical significance of our screening method. Second, we only used the MoCA to measure cognitive performance rather than performing a comprehensive assessment of each domain of cognition. The studies utilizing a formal neuropsychological test battery are needed to confirm our results. Further, the longitudinal studies are needed to confirm the results of this cross-sectional study.

In conclusion, this study showed that the SCC-related factors differ between the patients with EOPD and LOPD. Better visuospatial/executive abilities and memory measured by MoCA are related to a better self-reported cognitive status in EOPD, while older age at onset and apathy are related to SCCs in EOPD. Moreover, apathy and anxiety are associated with SCCs in LOPD. These results reveal that SCCs may reflect subthreshold cognitive impairments in the patients with EOPD who do not reach the diagnosis of MCI or PDD. SCCs are associated only with mood disorders in the patients with LOPD. More research should be conducted to investigate the prevalence and characteristics of SCCs in the EOPD and LOPD groups.

## Data Availability Statement

The original contributions presented in the study are included in the article/Supplementary Material, further inquiries can be directed to the corresponding author/s.

## Ethics Statement

The studies involving human participants were reviewed and approved by Ethics Committee of West China Hospital of Sichuan University. The patients/participants provided their written informed consent to participate in this study.

## Author Contributions

YX: organization and execution of research project, design and execution of statistical analysis, writing of the draft, and review and critique of the manuscript. RO: organization and execution of research project, design and execution of statistical analysis, and review and critique of the manuscript. TY and KL: organization and execution of research project and review and critique manuscript. QW, YH, LZ, and JL: organization and execution of research project. HS: conception, organization and execution of research project, and review and critique of the manuscript. All authors contributed to the article and approved the submitted version.

## Funding

This work is funded by the National Key Research and Development Program of China (Grant No. 2016YFC0901504), the 1.3.5 project for disciplines of excellence, West China Hospital, Sichuan University (Grant No. ZY2016203), the National Science Fund of China (Grant No. 81901293), and the Science Foundation of Chengdu Science and Technology Bureau (2019-YF05-00307-SN).

## Conflict of Interest

The authors declare that the research was conducted in the absence of any commercial or financial relationships that could be construed as a potential conflict of interest.

## Publisher's Note

All claims expressed in this article are solely those of the authors and do not necessarily represent those of their affiliated organizations, or those of the publisher, the editors and the reviewers. Any product that may be evaluated in this article, or claim that may be made by its manufacturer, is not guaranteed or endorsed by the publisher.
