# Supplementary material for: Different Associated Factors of Subjective Cognitive Complaints in Patients With Early- and Late-Onset Parkinson's Disease
Source: Front Neurol. 2021 Nov 23;12:749471. doi: 10.3389/fneur.2021.749471 (PMC8650060; doi:10.3389/fneur.2021.749471)
Supplement: Supplementary file 1 [file Data_Sheet_1.docx]

| **Supplementary Table 1 Factors associated with SCC in PD patients in univariate logistic regressions.** | | | |
| --- | --- | --- | --- |
|  | **OR** | **95%CI** | **p-value** |
| **Demographic features** |  |  |  |
| **Age(years)** | 1.02 | 1.00-1.05 | 0.024 |
| **Male** | 1.05 | 0.63-1.76 | 0.858 |
| **Age at onset(years)** | 1.02 | 1.00-1.05 | 0.028 |
| **Disease duration(years)** | 1.07 | 0.87-1.31 | 0.527 |
| **Education(years)** | 0.97 | 0.90-1.04 | 0.331 |
| **MDS-UPDRS Ⅲ** | 1.04 | 1.01-1.06 | 0.004 |
| **H&Y stage** | 2.24 | 1.29-3.92 | 0.004 |
| **BMI** | 1.07 | 0.98-1.16 | 0.115 |
| **Smoking** | 0.80 | 0.44-1.47 | 0.474 |
| **Drinking** | 0.39 | 0.20-0.76 | 0.006 |
| **Non-motor symtoms** |  |  |  |
| **MoCA** | 0.67 | 0.53-0.84 | ＜0.001 |
| **Visuospatial/executive abilities** | 0.77 | 0.56-1.06 | 0.109 |
| **Naming** | 1.13 | 0.61-2.10 | 0.699 |
| **Attention** | 0.75 | 0.43-1.33 | 0.328 |
| **Language** | 0.93 | 0.59-1.46 | 0.747 |
| **Abstraction** | 0.75 | 0.48-1.15 | 0.188 |
| **Memory** | 0.69 | 0.53-0.89 | 0.004 |
| **Orientaion** | 0.68 | 0.32-1.44 | 0.311 |
| **FSS** | 1.03 | 1.02-1.05 | ＜0.001 |
| **PDSS-2** | 1.07 | 1.03-1.11 | ＜0.001 |
| **RBD** | 1.65 | 0.83-3.28 | 0.156 |
| **LARS** | 1.09 | 1.05-1.12 | ＜0.001 |
| **HAMD** | 1.12 | 1.08-1.17 | ＜0.001 |
| **HAMA** | 1.12 | 1.07-1.17 | ＜0.001 |
| **ESS** | 1.03 | 0.97-1.09 | 0.327 |
| **Orthostatic hypotension** | 1.66 | 0.61-4.54 | 0.321 |
| PD: Parkinson's disease. SCC: subjective cognitive complaints. H&Y stage: Hoehn and Yahr stage. MDS-UPDRS-Ⅲ: Movement Disorder Society Unified Parkinson’s Disease Rating Scale part III. BMI: body mass index. MoCA: Montreal Cognitive Assessment. HAMD: Hamilton Depression Rating Scale. HAMA: Hamilton Anxiety Rating Scale. FSS: Fatigue Severity Scale. LARS: Lille Apathy Rating Scale.PDSS-2: Parkinson's Disease Sleep Scale 2nd version. EDS: Excessive daytime sleepiness. RBD: rapid eye movement sleep behavior disorder. BMI: body mass index. | | | |

| **Supplementary Table 2 Correlative clinical factors of SCC in PD patients.** | | | |
| --- | --- | --- | --- |
|  | **OR** | **95%CI** | **p-value** |
| **Drinking** | 0.37 | 0.18-0.78 | 0.009 |
| **Age at onset** | 1.03 | 1.00-1.05 | 0.029 |
| **Memory** | 0.66 | 0.49-0.88 | 0.005 |
| **LARS** | 1.05 | 1.01-1.09 | 0.013 |
| **HAMD** | 1.09 | 1.04-1.15 | 0.001 |
| PD: Parkinson's disease. SCC: subjective cognitive complaints. HAMD: Hamilton Depression Rating Scale. LARS: Lille Apathy Rating Scale. | | | |
